# Integrative transcriptomic and metabolomic analysis of D-leaf of seven pineapple varieties differing in N-P-K% contents

**DOI:** 10.1186/s12870-021-03291-0

**Published:** 2021-11-22

**Authors:** Jing Chen, Hui Zeng, Xiumei Zhang

**Affiliations:** 1 Key Laboratory of Tropical Fruit Tree Biology, Ministry of Agriculture, Zhanjiang, Guangdong 524091 China; 2 South Subtropical Crops Research Institute, Chinese Academy of Tropical Agricultural Sciences, Zhanjiang, Guangdong 524091 China

**Keywords:** Ca^2+^ dependent K^+^ transport, D-leaf, GS/GOGAT, K^+^ transport, Leaf color, N assimilation, NPK assimilation, N transport, Phosphate transporters, Photosynthesis

## Abstract

**Background:**

Pineapple (*Ananas comosus* L. Merr.) is the third most important tropical fruit in China. In other crops, farmers can easily judge the nutritional requirements from leaf color. However, concerning pineapple, it is difficult due to the variation in leaf color of the cultivated pineapple varieties. A detailed understanding of the mechanisms of nutrient transport, accumulation, and assimilation was targeted in this study. We explored the D-leaf nitrogen (N), phosphorus (P), and potassium (K) contents, transcriptome, and metabolome of seven pineapple varieties.

**Results:**

Significantly higher N, P, and K% contents were observed in Bali, Caine, and Golden pineapple. The transcriptome sequencing of 21 libraries resulted in the identification of 14,310 differentially expressed genes in the D-leaves of seven pineapple varieties. Genes associated with N transport and assimilation in D-leaves of pineapple was possibly regulated by nitrate and ammonium transporters, and glutamate dehydrogenases play roles in N assimilation in arginine biosynthesis pathways. Photosynthesis and photosynthesis-antenna proteins pathways were also significantly regulated between the studied genotypes. Phosphate transporters and mitochondrial phosphate transporters were differentially regulated regarding inorganic P transport. WRKY, MYB, and bHLH transcription factors were possibly regulating the phosphate transporters. The observed varying contents of K% in the D-leaves was associated to the regulation of K^+^ transporters and channels under the influence of Ca^2+^ signaling. The UPLC-MS/MS analysis detected 873 metabolites which were mainly classified as flavonoids, lipids, and phenolic acids.

**Conclusions:**

These findings provide a detailed insight into the N, P, K% contents in pineapple D-leaf and their transcriptomic and metabolomic signatures.

**Supplementary Information:**

The online version contains supplementary material available at 10.1186/s12870-021-03291-0.

## Background

Pineapple (*Ananas comosus* L. Merr.) is China’s third most important tropical fruit, which is cultivated on about 70,000 hectares. In 2019, China ranked 5th in terms of pineapple production with 1727 thousand metric tons (www.statistica.com). Since the start of a special research program in 2006 in order to accelerate the developments in pineapple industry. One of the key research objectives is the plant nutrient and fertilizer management [1]. Studies have shown that the uptake of nitrogen (N), phosphorus (P), and potassium (K) per day differs in different cultivars being cultivated in China. For example, Smooth Cayenne can uptake nearly 1/3rd more NPK as compared to Comte de Paris indicating different needs of fertilizers [1]. The differences in NPK contents are directly associated with the plant growth and development and fruit quality in different species. In apple, fruit quality, yield, and leaf micronutrients content varied with the applied NPK fertilizers [2]. The applied fertilizers can directly influence the nutrient concentrations in D-leaf of pineapple, which ultimately have positive influence on the yield and fruit size [3]. Particularly, the leaves either directly receive the sprayed nutrients or roots absorb the nutrients from soil, which are then translocated into the leaves and assimilated. Also, the leaf NPK contents directly influence the leaf photosynthetic capacity and thus affects overall growth and development and reproductive development in pineapple [4, 5].

Once absorbed by the plant roots in the form of ammonia or nitrate with the help of ammonium transporters (AMTs) and nitrate transporters (NRTs), N is transported to different plant organs and tissues. Once arrived it is assimilated through GS/GOGAT cycle (glutamine synthetase (GS) and glutamate synthase (GOGAT)) as well asparagine synthetase (AS), carbamoylphosphate synthase (CPsase), and mitochondrial glutamate dehydrogenase (GDH) [6]. Being prominent site of N assimilation, the leaf N content also depends on photosynthesis, carbon (C) metabolism, and amino acid synthesis [7]. Multiple studies have attempted to measure the differential N contents in different pineapple varieties cultivated across the globe [1, 4, 8, 9], however, the pathways and their differential regulation in this fruit plant have not been discovered in detail. Since, plants take up numerous nutrients (majorly N and P) from soil, which are also transported and assimilated in leaves as well as other plant tissues [10]. Like N transporters, phosphate (inorganic phosphate, Pi) is also transported from soil to the plants with the help of phosphate transporters (PHT) [11]. Other than PHTs, vacuolar phosphate transporters (VPTs), Pi/H^+^ antiporters also serve as Pi influx transporters into the vacuolar lumen [12, 13]. Pi is not only stored in vacuole but also in chloroplasts and mitochondria. Several members of PHT family are localized in these two organelles e.g., PHT2, PHT3, and PHT4 [14, 15]. These PHTs are post-transcriptionally regulated by a number of a number of genes and transcription factors (TFs) including actin related protein 6 (ARP6), inositol pentakisphosphate 2 kinase 1 (IPK1), induced by phosphate starvation 1 (IPS1), WRKYs, MYB-type, and bHLH TFs [15–17]. Along with the N and P, K^+^ plays key roles in many processes in living cells and is considered indispensable for plant growth. K^+^ is taken up by plant roots by Arabidopsis K^+^ transporter 1 (AKT1) in a coordinated manner with calcineurin B-like (CBLs) and their interacting kinases (CIPKs) [18]. Apart from these, high affinity K^+^ transporter 5 (HAK5), and K^+^ uptake transporters (KUPs) are also a major transporter [19–21]. The root-absorbed-K^+^ is translocated to aerial parts of the plants via xylem vessels. In this process, Stellar K^+^ Outward Rectifier (SKOR) and Guard Cell Outwardly Rectifying K^+^ channels (GORK) belonging to shaker family K^+^ channels play major roles [22, 23]. Once arrived in the leaves, K^+^ is stored mostly stored in vacuole, where its sequestration is mediated by cation/H^+^ antiporters (NHXs) [24, 25] and the K^+^ release (efflux) from vacuole is driven by the tandem por K^+^ channels (TPKs) [26]. Studies have also confirmed the involvement of Ca^2+^ signaling related genes in K+ efflux and sequestration [27]. Apart from the selective transport and assimilation related genes and TFs, the leaf NPK contents have also been studied to be affected by cytokinins and auxin signaling. For example, in pineapple, it was reported that the endogenous levels of the phytohormones was significantly different in plant grown under medium with or without N [28]. However, a complete understanding on the above-mentioned transport and assimilation related genes, TFs, and pathways is scarce. Particularly, the existing varieties must be explored in order to develop a nutrition management strategy for individual varieties.

The need for NPK nutrition in plants can be easily detected from the leaf colour as in case of rice, maize, soybean, and other crops [29–31]. However, the famous cultivated varieties in China i.e., BL, CN, Golden pineapple (MD-2), and Tainong varieties (TN) were observed to have different leaf colors i.e., ranging from dark green to purple-green (See Methods section for details). Such a variation makes it difficult for farmers to estimate the NPK requirements of the plant, which is an easy task in other crop plants [29–31]. To this regard, there is very limited information available on the differential NPK contents in the leaves of the pineapple varieties that are being cultivated in China. Furthermore, there is no information available on the differential transcriptome and metabolomes in these varieties. Here, we attempted to check the NPK% contents in the seven pineapple varieties i.e., MD-2, CN, BL, TN4, TN17, TN21, and TN23, followed by transcriptome and metabolome profiling. In this study, we discussed the differential expression of key genes in transport and assimilation of N, P, and K in D-leaves of the selected pineapple variety. Furthermore, we also provide insights into the accumulated metabolites in the D-leaves of the studied pineapple varieties.

## Results

### Variation in D-leaf N, P, and K contents

**Fig. 1:**
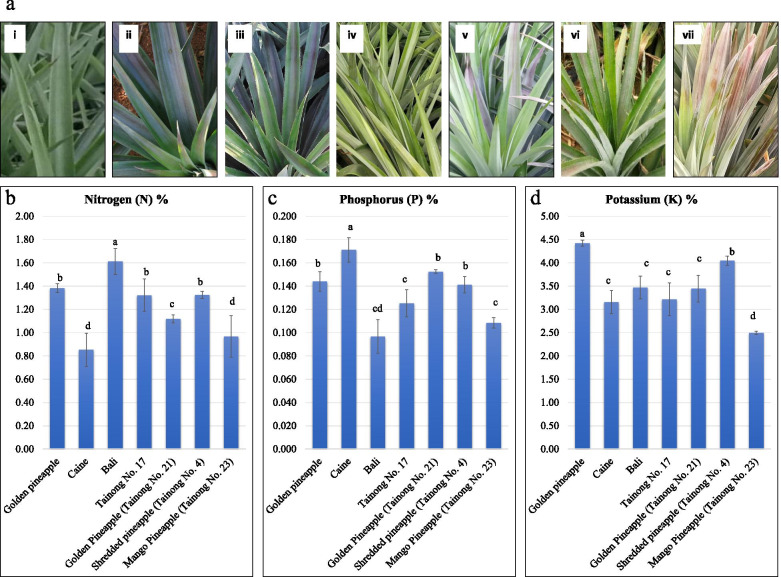
**a)** Leaf color variation in seven pineapple varieties i.e., (i) Golden pineapple (MD-2), (ii) Caine (CN), (iii) Bali (BL), (iv) Tainong no. 21 (TN21), (v) Tainong no. 17 (TN17), (vi) Tainong no. 4 (TN4), and (vii) Tainong no. 23 (TN23). **b)** D-leaf nitrogen, **c)** phosphorus, and **d)** potassium percent contents in seven pineapple varieties. X-axes represent the tested varieties and Y-axis represent the % content of the nutrient. Different letters on the bars show that the varieties differ significantly for the nutrient percent content (p < 0.05)

Phenotypic appearance of the seven pineapple varieties clearly indicates that the leaves differ in their color appearance (Fig. 1a). Considering these differences, the N, P, and K % contents in D-leaves were measured (Fig. 1b-d). Bali (BL) had significantly higher N% among all the seven varieties followed by Golden pineapple (MD-2), Tainong-17 (T17), Tainong-4 (T4), Tainong-21 (T21), Tainong-23 (T23), and Caine (CN, also known as Smooth Cayene) (Fig. 1b). CN had significantly higher P contents, while MD-2, T21, and T4 had similar P contents. Lowest P contents were recorded in BL and T23 (Fig. 1c). Highest and lowest K contents were observed in MD-2 and T4, respectively, while CN, BL, T17, and T21 had no significant differences. Second highest K content was measured in T4 (Fig. 1d). When summed up, the overall best N+P+K % contents were highest in MD-2, followed by T4, BL, T21, T17, CN, and T23 (Supplementary Fig. 1 ). These observations suggest that BL, CN, and MD-2 are best varieties from individual D-leaf nutrient content perspective.

### Transcriptome sequencing of D-leaves of seven pineapple varieties

**Fig. 2:**
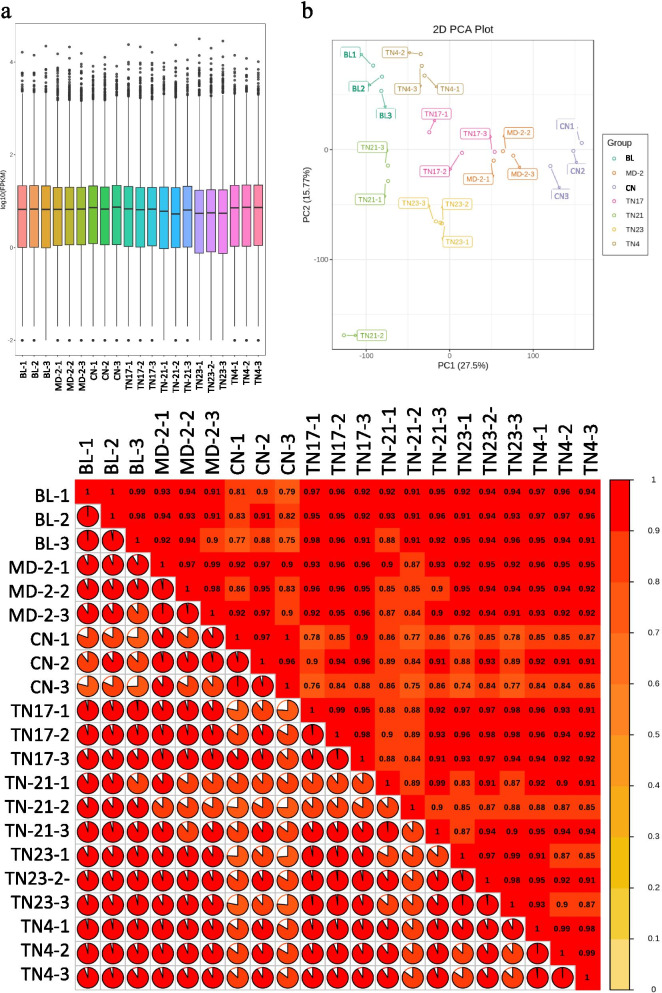
Quantification of gene expression in D-leaves of seven pineapple verities. **a** Expressive bar plot representing overall distribution of gene expression. **b** Principal component analysis 2D plot. **c** Pearson Correlation Coefficients between biological replicates represented as a heatmap. Golden pineapple (MD-2), Caine (CN), Bali (BL), Tainong no. 4 (TN4), Tainong no. 17 (TN17), Tainong no. 21 (TN21), and Tainong no. 23 (TN23)

The transcriptome of 21 samples (three replicates of each variety) was sequenced using Illumina HiSeq High-throughput sequencing platform. Clean read ranging from 44.82 to 80.08 million/sample (on average 54.44 million reads) were obtained from 21 Illumina libraries (Supplementary Table 1 ). A total of 169.1 Gb of clean data was obtained. The Q20 and Q30 base %, and GC content % was 97.9 %, 94.09 %, and 50.71 %, respectively. Overall, on an average 91.54 % reads could be mapped to the reference genome. Overall, FPKM values of CN, BL, and TN4 were higher than other varieties (Fig. 2a). The replicates of each variety tended to group together (Fig. 2b). Furthermore, the average PCC between the replicates and treatments was > 0.8 (Fig. 2c). These observations support that the expression results are reliable and have least variability between the replicates of each variety.

### Differential Gene Expression between seven pineapple varieties

**Fig. 3:**
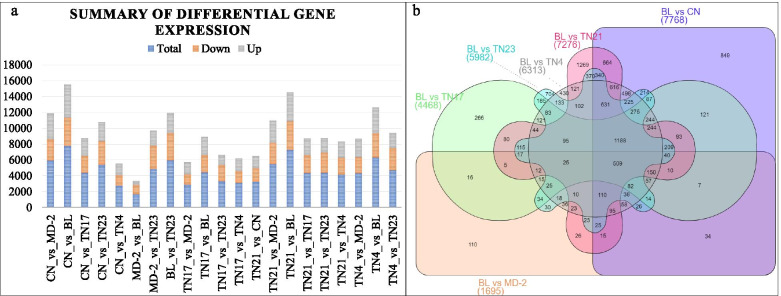
**a** Summary of the differential gene expression in different pineapple variety comparisons. **b** Venn diagram showing the number of DEGs between BL versus other six pineapple varieties. Golden pineapple (MD-2), Caine (CN), Bali (BL), Tainong no. 4 (TN4), Tainong no. 17 (TN17), Tainong no. 21 (TN21), and Tainong no. 23 (TN23)

In order to screen the differentially expressed genes (DEGs), we used a criterion i.e., |log2 Fold Change| >= 1, and FDR <0.05. These screening conditions resulted in the identification of 14,310 DEGs in the studied pineapple D-leaves (Supplementary Table 2 ; Fig. 3a).

### D-leaf transcriptome comparison of BL with other varieties

Since the D-leaves of the BL variety had significantly high N% contents, therefore, we compared the gene expression of BL with the rest of the varieties and completed subsequent results. There were 1695, 7768, 4468, 6313, 7276, and 5982 DEGs in BL vs. MD-2, BL vs. CN, BL vs. TN17, BL vs. TN4, BL vs. TN21, and BL vs. TN23, respectively (Fig. 3b). The DEGs were significantly enriched in different KEGG pathways associated with signaling (MAPK signaling), photosynthesis, photosynthesis-antenna proteins, and plant-pathogen interaction. Furthermore, the DEGs were also enriched in pathways associated with nitrogen mobilization and assimilation (see details below).

### Differential regulation of N transport, assimilation and related pathways

**Fig. 4:**
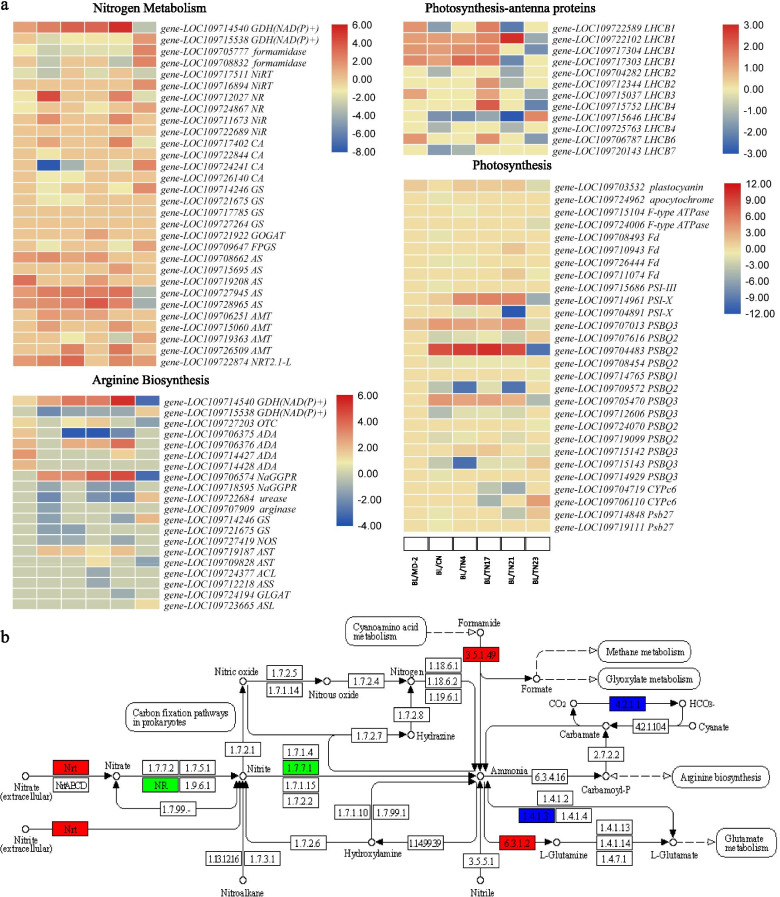
Differentially expressed genes involved in nitrogen assimilation. Heatmaps represent the DEGs enriched in (**a**) nitrogen metabolism, arginine biosynthesis, photosynthesis-antenna proteins, and photosynthesis pathways. **b** Differential regulation of nitrogen metabolism pathway between BL and CN pineapple varieties. Red, green, and blue indicate the DEGs that were upregulated, downregulated and up/downregulated in BL as compared to CN, respectively. Golden pineapple (MD-2), Caine (CN), Bali (BL), Tainong no. 4 (TN4), Tainong no. 17 (TN17), Tainong no. 21 (TN21), and Tainong no. 23 (TN23)

Nitrogen is assimilated by GS/GOGAT cycle [32]. In this cycle several key players take part such as GS and GOGAT. Additionally, AS, CPSase, and GDH [6]. In the BL vs. other pineapple varieties comparisons, we observed the differential regulation of 30 genes that were associated with nitrogen assimilation pathway (Fig. 4a). First of all, we found that NRTs and AMTs were differentially expressed. The AMTs were not differentially expressed between BL and MD-2. Similarly, only one of four AMTs were differentially expressed between BL vs. TN17 as well as BL vs. TN23. In case of BL vs. CN, only two AMTs were differentially expressed. A high-affinity NRT-like (NRT2.1-L) showed increased expression in all pineapple varieties as compared to BL, while it didn’t differentially express in BL vs. TN17. These observations suggest that the arrival of N in the pineapple D-leaves differ in different varieties and is largely dependent on the expression of the AMTs and NRTs. We noticed that nitrate reductases (NAD(P)H) (NRs) had variable expression in different varieties i.e., their expression was lower in MD-2 and TN17, whereas higher in CN and TN21. Regarding N metabolism and assimilation, glutamate dehydrogenase (GDH), formamidase, nitrate reductase (NR), nitrite reductase (NiR), carbonic anhydrase (CA), GS, GOGAT, and asparagine synthase (AS) were differentially expressed in the D-leaves of the pineapple varieties (Fig. 4a and b). Specifically, four GS and a GOGAT showed different FPKM values in different pineapple varieties. These genes were not differentially expressed in BL vs. MD-2 and BL vs. TN17. GS had higher expression in CN, TN4, and TN21 as compared to BL. Contrastingly, TN23 had lower GS FPKM values. GOGAT was only differentially expressed in BL vs. TN17; higher expression in TN17 as compared to BL. These observations suggest that GS/GOGAT are not key players in pineapple D-leaf N content variation. Two GDH(NAD(P)+) had contrasting expression patterns in the BL vs. other variety comparisons; one (*GDH2*; *gene-LOC109714540*) was upregulated in all (except TN23) as compared to BL, while the second (*GDH1*; *gene-LOC109715538*) had contrasting expression. Thus, the variable N contents might be under the influence of both of these genes. Both GDHs play role in inducing changes in the contents of C and N as well as of starch/glucose and glutamine [33]. Since GDHs are known for their roles in arginine biosynthesis as well as N assimilation, therefore, we further searched for the DEGs enriched in arginine biosynthesis pathway. We found 20 DEGs enriched in arginine biosynthesis pathway, including both GHDs (Fig. 4a). Other DEGs included GSs, nitric oxide synthase (NOS), acetylornithine deacetylases (ADAs), N-acetyl-gamma-glutamyl-phosphate reductase (NaGGPR), aspartate aminotransferases (ASTs), and glutamate N-acetyltransferase (GLGAT). The ADAs had expressions in MD-2 and TN21, while none was differentially expressed in BL vs. TN23.

Additionally, we found 12 antenna proteins (LHCBs, light-harvesting complex II chlorophyll a/b binding proteins), and 28 DEGs associated with photosynthesis pathway (Fig. 4a). The LHCBs showed variable expression patterns in different pineapple varieties. For example, all LHCBs had higher expressions in MD-2 as compared to BL. LHCB4s were mostly downregulated in pineapple varieties as compared to BL. All LHCBs were upregulated in TN17 as compared to BL. The genes annotated as Fd, were not differentially expressed in all variety comparisons; downregulated in CN, TN4, and TN23, and upregulated in TN21. Similarly, F-type ATPases were only differentially expressed in BL vs. TN4 and BL vs. TN23; where BL had higher expressions. Major expression changes were noted in photosystem II oxygen-evolving enhancer proteins (PSBQs). PSBQ1 was only differentially expressed in BL vs. TN23 (downregulated in TN23). PSBQ2s and PSBQ3s showed both up/downregulation in the compared varieties to BL. One particular observation was the contrasting regulation of the PSBQ protein in TN23. When these proteins were upregulated in other varieties, TN23 showed downregulation and vice versa. This was also true for PSI subunit x, cytochrome c6, and Psb27 (Fig. 4a).

### D-leaf transcriptome comparison of CN with other varieties

Considering higher P% in CN pineapple, we sought to compare its D-leaf transcriptome with the remaining six varieties. There were 5951, 7768, 2761, 4383, 3263, and 5404 DEGs in CN vs. MD-2, CN vs. BL, CN vs. TN4, CN vs. TN17, CN vs. TN21, and CN vs. TN23, respectively (Supplementary Fig. 2 a). The DEGs were significantly enriched in different pathways i.e., plant-pathogen interaction, starch and sucrose metabolism, flavonoid biosynthesis, phenylpropanoid biosynthesis, ABC transporters, and glutathione metabolism.

### Differential regulation of genes associated with P transport and metabolism

**Fig. 5:**
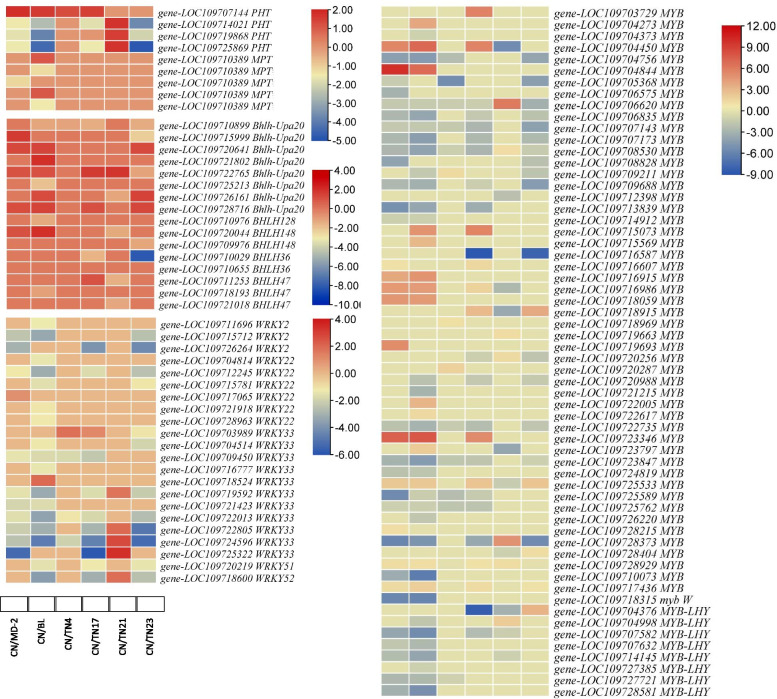
Heatmaps of DEGs related to phosphate transport in D-leaves of six pineapple varieties as compared to CN. Golden pineapple (MD-2), Caine (CN), Bali (BL), Tainong no. 4 (TN4), Tainong no. 17 (TN17), Tainong no. 21 (TN21), and Tainong no. 23 (TN23)

Phosphorus plays vital roles in plants including energy transfer, photosynthesis, transformation of sugars and starches, and is a major component of nucleic acids, membrane lipids, and phosphorylated intermediates of energy metabolism [34–36]. We observed the differential expression of nine P transporters (Fig. 5). Of these, four were inorganic phosphate transporters (PHTs); only one (*gene-LOC109707144*) was upregulated in MD-2, BL, TN4, and TN17 as compared to CN, while three others were downregulated in other genotypes as compared to CN. Remaining five P transporters were members of solute carrier family 25 and annotated as mitochondrial phosphate transporters (MPTs). Interestingly, these MPTs were only differentially regulated between CN vs. MD-2 and CN vs. BL. Apart from this we also found that 22 WRKY transcription factors (TFs); two WRKY2, six WRKY22, 11 WRKY33, one WRKY51-like, and one WRKY52, were differentially regulated between the CN and other genotypes. The expression of these WRKYs was higher in MD-2, BL, TN4, TN17, and TN23 as compared to CN. Only, TN21 had higher expression of WRKYs (WRKY33s and WRKY52) as compared to CN. Suggesting that the P levels might be affected by changes in the WRKYs’ expression [37, 38]. We also checked if bHLH TFs, and MYB TFs were differentially regulated between these variety comparisons. Sixteen bHLH TFs and 60 MYB TFs were differentially expressed in D-leaves of different varieties. Eight MYB TF were annotated as MYB-related TF LHY; all of these were downregulated in all varieties except TN23, where only one MYB-LHY was differentially expressed (upregulated). Other MYB TFs showed variable expression patterns. All differentially expressed bHLH TF in CN vs. MD-2 were upregulated in MD-2 as compared to CN. Only one bHLH TF was differentially expressed in TN4; the expression was reduced as compared to CN, while others had variable expression in D-leaves of the tested varieties. These expression changes indicate that major players in Pi transport in D-leaf of pineapple are PHTs, MPTs, WRKYs, MYBs, and bHLHs [16, 17] (Fig. 5).

### D-leaf transcriptome comparison of MD-2 with other pineapple varieties

Transcriptome comparison of MD-2 with the remaining six pineapple varieties revealed that there were 5951, 1695, 4351, 2876, 5488, and 4861 DEGs in MD-2 vs. CN, MD-2 vs. BL, MD-2 vs. TN4, MD-2 vs. TN17, MD-2 vs. TN21, and MD-2 vs. TN23, respectively. These DEGs were significantly enriched in plant-pathogen interaction, ABC transporters, starch and sucrose metabolism, glutathione metabolism, anthocyanin biosynthesis, and flavonoid biosynthesis pathways.

### Differential regulation of genes associated with K transport and metabolism

**Fig. 6:**
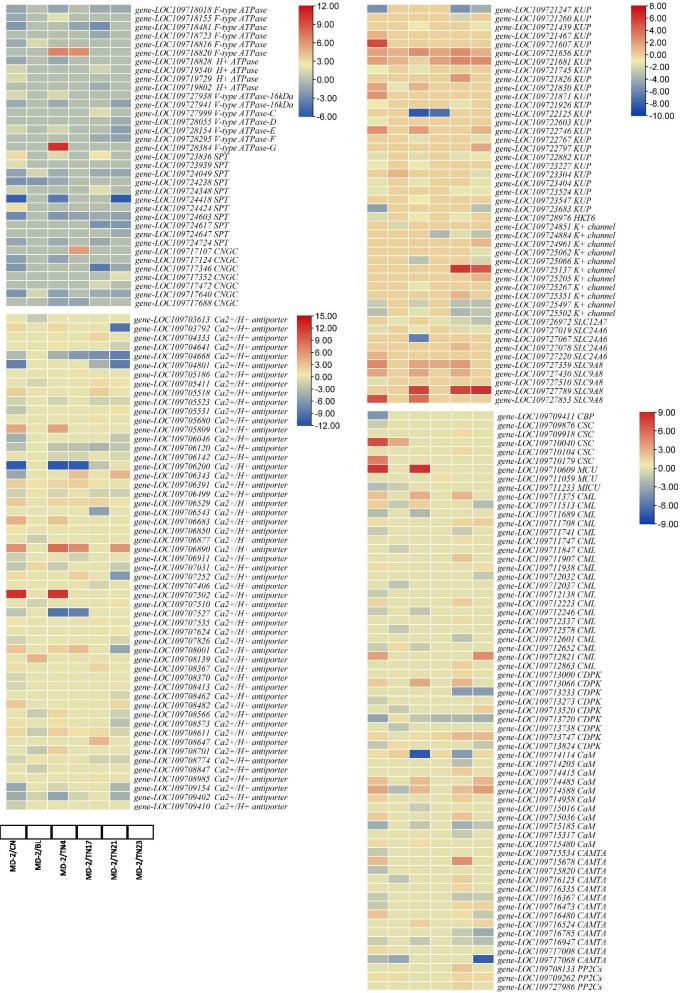
Heatmaps of the differentially expressed genes related to K^+^ transport and assimilation in D-leaves of MD-2 as compared to the other pineapple varieties. Golden pineapple (MD-2), Caine (CN), Bali (BL), Tainong no. 4 (TN4), Tainong no. 17 (TN17), Tainong no. 21 (TN21), and Tainong no. 23 (TN23)

Upon arrival in the roots, K^+^ is either stored in vacuoles locally or transported to the aerial parts where it is accumulated. To this regard, we found the differential regulation of four H^+^ transporting ATPases. Three were differentially regulated in MD-2 vs. CN, where their expression was lower in CN. None was regulated between MD-2 vs. BL, TN17, and TN23. One was expressed in TN4 (upregulated), while two were expressed variedly in TN21 (Fig. 6). Apart from these, we also found the differential expression of F-type H^+^ transporting ATPases between the variety comparisons. Both types of H^+^ transporting ATPases play roles in K^+^ transport in plants [39]. Seven V-type H^+^-transporting ATPases (two 16 kDa proteolipid subunits, subunits c, d, e, f, and g) were differentially expressed in pineapple D-leaves. One 16 kDa proteolipid subunit was upregulated in CN and TN4, while the other was downregulated in CN, TN4, and TN23. Subunit C was upregulated in TN21, subunit d was upregulated in TN23, subunit e was upregulated in CN, TN4, while downregulated in TN23, subunit F was downregulated in TN21 and TN23, and subunit G was upregulated in TN4, respectively. These expression changes along with the regulation of 11 sugar:H^+^ symporters (SPTs), and members of solute carrier families 12, 24, and 9 (SLCs) is indicative of differential K% content within the D-leaves of the studied pineapple varieties (Fig. 6). Major observation was the differential expression of 24 KUP system potassium uptake proteins (KUPs). Seven and eight KUPs were up- and downregulated in CN as compared to MD-2, respectively. In MD-2 vs. BL, MD-2 vs. TN4, MD-2 vs. TN17, MD-2 vs. TN21, MD-2 vs. TN23 there were 4, 11, 3, 7, and 11 differentially regulated KUPs. We observed that the expression of some KUPs was increased while in MD-2 while others’ expression was decreased. We also found differential regulation of K-channels in D-leaves of the compared varieties. Maximum number of K-channels were differentially expressed between MD-2 and TN23 (eight) followed by MD-2 vs. TN21 (five). Other variety comparisons showed the expression of four or less K-channels (Fig. 6). Interestingly, we found that MD-2 K-channels had higher expression as compared to all varieties except three and two genes in TN21 and TN23, respectively. Considering the higher expression of these channels as well as the differential expression of seven cyclic nucleotide gated channels (CNGCs), it could be suggested a key player in higher K% contents in MD-2. We also found one probable cation transporter HKT6, which was downregulated in CN, TN4, and TN21 as compared to MD-2. Apart from transporters, we also looked for genes that affect the expression of K-transporters and related genes. Interestingly, a large number of Ca^2+^ exchange, transport, and signaling related genes were differentially expressed in D-leaves of studied pineapple varieties. Forty-nine Ca^2+^/H^+^ antiporters, five Ca^2+^ transporting ATPases, a Ca^2+^ binding protein 39, 5 calcium permeable stress-gated cation channels, 2 Ca^2+^ uniporter proteins, 1 Ca^2+^ uptake protein 1, 20 calcium-binding protein CMLs (CMLs), 9 calcium-dependent protein kinases (CDPKs), 11 calmodulins (CaMs), three protein phosphatase 2Cs (PP2Cs) and 13 calmodulin-binding transcription activators (CAMTAs) (Fig. 6).

### Quantitative Real-Time PCR analysis

**Fig. 7:**
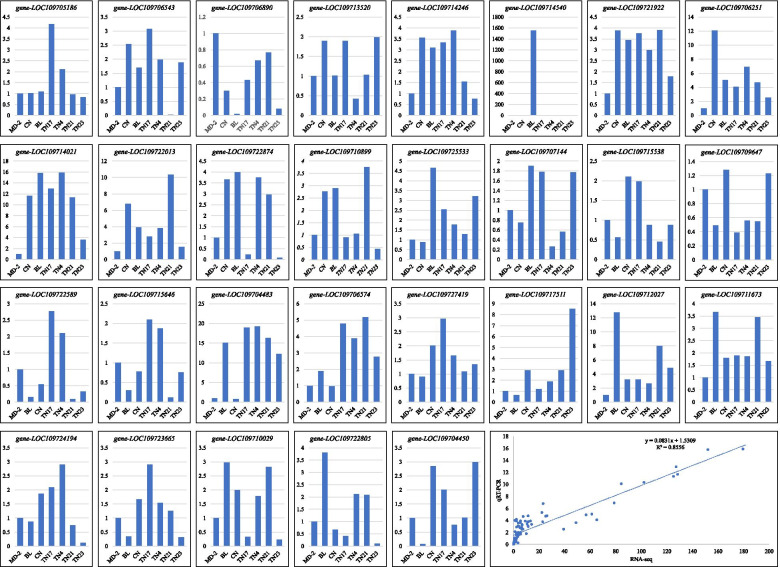
Expression analysis of selected genes in D-leaves of seven pineapple varieties. X-axes represent the varieties and Y-axes represent the relative gene expression. The last panel shows correlation between qRT-PCR expression data and RNA-Seq data of the selected genes. Golden pineapple (MD-2), Caine (CN), Bali (BL), Tainong no. 4 (TN4), Tainong no. 17 (TN17), Tainong no. 21 (TN21), and Tainong no. 23 (TN23). X-axis shows varieties and Y-axis shows relative gene expression

We validated the expression of 29 pineapple genes of interest (Fig. 7). The *Actin* gene was used as an internal control. Based on the correlation (R^2^ = 0.8556) between qRT-PCR data and RNA-seq data of the genes, we found that the expression of the selected genes was in accordance with the RNA-seq data (Fig. 7). These analyses validate the transcriptome sequencing results and confirm their reliability.

Apart from the validation of the reliability of the RNA sequencing data, the genes also show that the studied varieties express these genes differentially as discussed in the above sections.

### Metabolite profiles of D-leaves of pineapple varieties

**Fig. 8:**
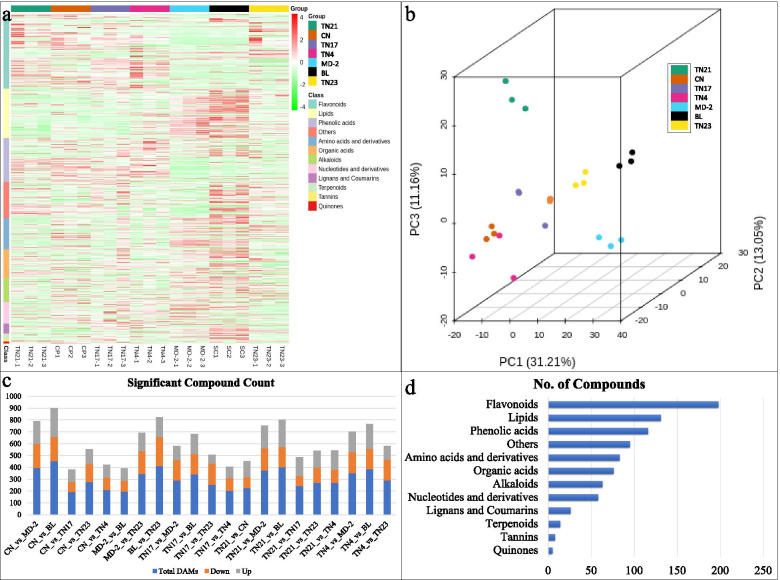
Metabolite profile of D-leaves of pineapple varieties. **a** Heatmap showing the intensities of the detected compounds and their classification, **b** principal component analysis, **c** significant compound count between the variety comparisons, and **d** number of detected compounds in each class. DAMs: Differentially accumulated metabolites. Golden pineapple (MD-2), Caine (CN), Bali (BL), Tainong no. 4 (TN4), Tainong no. 17 (TN17), Tainong no. 21 (TN21), and Tainong no. 23 (TN23)

UPLC-MS/MS detection platform, a self-built database, and the multivariate statistical analyses of 21 samples belonging to D-leaves of seven pineapple varieties resulted in the detection of 873 metabolites (Supplementary Table 3 ; Fig. 8a). The detected metabolites were broadly classified as flavonoids, lipids, phenolic acids, amino acids and derivatives, alkaloids, nucleotides and derivatives, lignans and coumarins, terpenoids, tannins, and quinones (Fig. 8a). The PCA analysis grouped the replicates according to the varieties. TN23, MD-2, and BL showed variability from other varieties, while the rest of varieties tended to group (Fig. 8b). The accumulated metabolites were screened based on fold change ≥ 2 and ≤ 0.5 and VIP ≥ 1; the differentially accumulated metabolites (DAMs) in the D-leaves of the seven pineapple varieties are shown in Fig. 8c. The D-leaves of pineapple varieties were rich in flavonoids (198 compounds), lipids (131 compounds), and phenolic acids (116 compounds) (Fig. 8d).

### Differentially accumulated metabolites in D-leaves of pineapple varieties

**Fig. 9:**
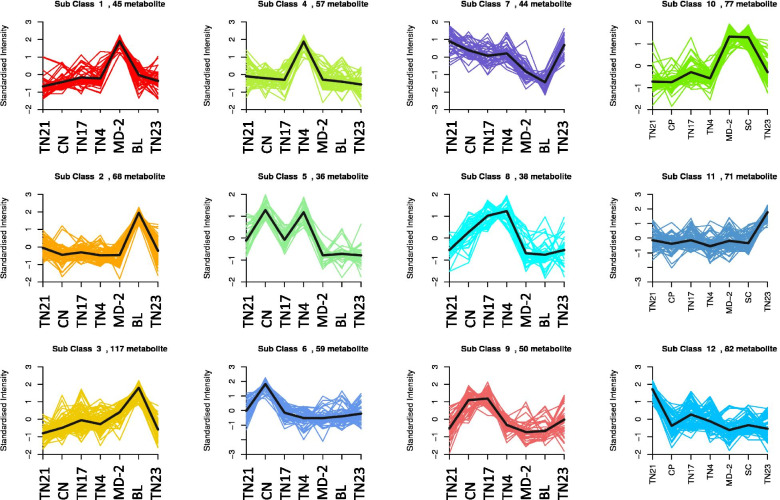
K-means cluster analysis of the detected metabolites in D-leaves of seven pineapple varieties. Golden pineapple (MD-2), Caine (CN), Bali (BL), Tainong no. 4 (TN4), Tainong no. 17 (TN17), Tainong no. 21 (TN21), and Tainong no. 23 (TN23)

In order to understand the accumulation of metabolites in pineapple varieties having higher N, P, and K% contents, we used the K-means cluster analysis results. As discussed above, the BL pineapple had significantly higher N% contents, therefore, we looked into the subclass of compounds that had higher accumulation in BL as compared to other varieties. Three subclasses of compounds i.e., subclass 2 (68 metabolites), 3 (117 metabolites), and 10 (77 metabolites) showed higher standardized intensities of compounds as compared to other varieties (Fig. 9). The metabolites in subclass 2 were mainly saccharides and alcohols, and organic acids. Subclass 3 had a higher number of metabolites classified as lipids, amino acids and derivatives, flavonoids, organic acids, and phenolic acids. Similarly, subclass 10 had also higher number of metabolites classified as lipids, amino acids and derivatives, lignans and coumarins, and flavonoids (Supplementary Table 4). The top-10 most accumulated metabolites in BL were Methylmalonic acid*, succinic acid, arachidic acid, L-valine, lysoPE 18:1, D-glucose, pipecolic acid, D-fructose, L-valyl-L-leucine, and 4-Nitrophenol. Mainly, these metabolites were enriched in starch and sucrose metabolism, biosynthesis of amino acids, and fatty acid biosynthesis and related pathways. Two metabolites i.e., L-glutamine and L-glutamic acid were enriched in nitrogen metabolism pathway, however, their content was highest in MD-2 and TN4, respectively.

Since CN had higher P% contents, therefore, we looked for compounds with higher standardized intensities (subclass 5 (36 metabolites), 6 (59 metabolites), and 9 (50 metabolites)) in this pineapple variety as compared to others (Fig. 9). The metabolites included in subclass 5 were classified as phenolic acids and flavonoids, while those included in subclass 6 were classified as flavonoids, amino acids and derivatives, alkaloids, phenolic acids, and organic acids. Similarly, subclass 9 had higher number of flavonoids and organic acids. Top-10 most accumulated metabolites in CN were arachidic acid, chlorogenic acid, 2,3-dihydroxy-3-methylbutanoid acid, L-valine, L-ascorbic acid, neochlorogenic acid, cryptochlorogenic acid, 5,4’-dihydroxy-3,6,7,3’-tetramethoxyflavone-4’-O-glucoside, N’,N’’-Diferuloylspermidine, and aurantio-obtusin-6-O-glucoside. These top-accumulated metabolites were enriched in phenylpropanoid biosynthesis, flavonoid biosynthesis, secondary metabolite biosynthesis, and valine and isoleucine biosynthesis pathways (Supplementary Tables 3, 4).

As MD-2 had higher K% contents, it had higher standardized intensities of subclass 1 and 10 having 45 and 77 metabolites, respectively (Fig. 9). The metabolites included in subclass 1 were classified as nucleotides and derivatives, lipids, and amino acids, and derivatives while those included in subclass 10 were classified as lipids, amino acids and derivatives, lignans and coumarins, and flavonoids. Top-10 metabolites accumulated in MD-2 were arachidic acid, guanosine, methylmalonic acid, succinic acid, 2,3-dihydroxyl-3-methylbutanoic acid, L-valyl-L-Leucine, L-valine, lysoPE 18:1, L-norleucine, and L-gycyl-L-phenylalanine. These metabolites were mostly enriched in pathways related to amino acid biosynthesis, citrate cycle, ABC transporters, and biosynthesis of secondary metabolites (Supplementary Tables 3, 4).

## Discussion

Pineapple being the third most important tropical fruit in China needs attention regarding improvements in nutrient management and finding candidate genes/pathways to be engineered in this regard. In different crop plants, the leaf color indicates the nutrient content level and farmers manage to supply the nutrients either by spraying the above ground parts of by adding fertilizers to the soil [29–31]. We have observed that the leaf colors of the pineapple varieties differ from each other (Fig. 1a) and hence, designing a leaf-colour chart to help the farmers is quite difficult. Alternate strategy would be the understanding of the key regulatory genes and pathways and manipulating them through gene modification and engineering techniques. An early understanding for such a task is to determine the key transcriptomic and metabolomic changes. Through this study, we have provided the transcriptomic and metabolomic signatures in the D-leaves of seven famous pineapple varieties cultivated in China.

### Nitrogen transport, assimilation, and metabolism in the D-leaves of pineapple

Early researches had determined that variation in leaf NPK contents leads to changes in overall plant health, yield, and fruit quality [3, 9]. The mechanisms behind the variable NPK contents could be a possible reason since it is known that higher allocation of N in leaves maximizes C gain in leaf by increasing photosynthesis [40, 41]. Our observations that 30 genes related to nitrogen assimilation were differentially regulated indicates that the studied varieties differently utilize their N transport and assimilation to manage their D-leaf N contents. Particularly, the differential expression of N-transporters suggested that N arrives in D-leaves of pineapple varieties differently (Fig. 4a). Here it is important to discuss that the AMTs and NRTs are located in roots to absorb nitrogen from soil in form of ammonium and nitrate, however, the expression of NRTs (e.g., *NRT1.2*, *NRT1.4*, *NRT1.6*, and *NRT2.7*) [6, 10] and AMTs (*TeAMT2;3a*, *LjAMT1;1*, *BnAMT1;2*, *LeAMT1;2*, *NtAMT1;3*, *PtrAMT2;2*) has also been confirmed in leaves of different plants [42,43 and references therein]. The arrived N in pineapple D-leaves was being reduced to ammonium as evident from the differential expression of [44]. Further, the N assimilation in the D-leaves of the pineapple varieties differed as we noted the differential expression of GSs. It is important to note that this step of N assimilation/metabolism was not differentially regulated between BL (variety having significantly higher N% contents) and MD-2 and TN17. Since, our results showed the differential regulation of *GDH1* and *GDH2* in the D-leaves of the seven pineapple varieties, therefore, it is possible that differences in N% content could be their varied expressions [33]. Because, both GDHs had contrasting expressions, thus, these two genes are prime targets for characterization in pineapple. GSs along with GDH assimilate nitrogen into amino acids [45, 46]. Enrichment of DEGs as well as DAMs in amino acid biosynthesis related pathways (Fig. 7d) is indicating that these enzymes are primarily playing roles in N assimilation (owing to their presence in the upstream of biosynthesis pathways of multiple amino acids e.g., arginine biosynthesis (Fig. 4a), leucine and isoleucine biosynthesis (Supplementary Tables 3 and 4). The detection of a higher number of amino acids and derivatives in BL strongly correlates with the higher N% contents. Not only GS but also no differential expression of GOGAT in BL vs. TN17 proposes that the variation of N content in the D-leaves of these two varieties is possibly being regulated at some other step.

Since the NR, NiR, and GOGAT have different reducing power requirements (it could be NADH or ferredoxin, Fd), therefore, these should be provided by the photosynthesis or related pathways. Additionally, GS needs ATP and amino acid biosynthesis (organic N) needs C skeletons, which are received as products of photosynthesis. Therefore, the varied expression of the DEGs enriched in photosynthesis-antenna proteins pathways (LHCBs) suggests that different levels of light energy are being captured, which is affecting the delivery of excitation energy to the photosynthesis [6, 47]. Apart from the antenna proteins, the observation that Fd genes were downregulated in CN, TN4, and TN23 and upregulated in TN21 as compared to BL (Fig. 4a) suggests that the reducing power requirements for NR, NiR, and GOGAT are being regulated differently in the studied pineapple varieties (at least in D-leaves). It is known that reducing power is essential for nitrate and nitrite reduction and is mainly supplied by the photosynthetic electron transfer chain; the requisite Fd is generated here [48]. The expression changes of other proteins i.e., PSBQs further suggest that the studied pineapple varieties exhibit differential regulation of photosystem II. Thus, the N% content differences in these varieties are being regulated from transport to assimilation. Furthermore, the role of affiliated pathways is not to be neglected. For example, arginine has the highest N to C ratio (40-50 % of the total N reserves) [49], therefore the regulation of arginine biosynthesis pathway related genes i.e., GSs, GDHs, NOS, ADAs, NaGGPR, ASTs, and GLGATs in D-leaves of the studied pineapple varieties is of interest (Fig. 4a). The increased expression of ASTs in MD-2 as compared to BL is interesting. It could suggest that relatively higher N is MD-2 is being directed to arginine biosynthesis as compared to BL. We say this because of the known role of AST in arginine biosynthesis pathway [50]. Taken together, N the studied varieties differ for their D-leaf N% contents and the transcriptome and metabolome changes suggest the N transport, assimilation, metabolism, and downstream pathways i.e., biosynthesis of amino acids is being regulated at different steps. Furthermore, the photosynthesis and antenna proteins also take part in N assimilation.

### Pi transport and metabolism in D-leaves of pineapple

Phosphorus is a crucial macronutrient because it is a structural constituent nucleic acids, phospholipids, and energy related molecules in plants i.e., ATP. It is also integral to photosynthesis and respiration [51]. Many studies revealed the role of Pi in pineapple plant growth, development, yield, and fruit quality [3, 52–54]. Phosphorus is transported in the plant tissues through PHTs. D-leaves of the studied pineapple varieties have two different types of P transporters i.e., PHTs and MPTs, which showed variable expression patterns in the studied pineapple varieties. Pi transport is being controlled differentially. PHTs acquire and distribute Pi among tissues and also help plant to manage Pi homeostasis [51]. On the other hand, the MPTs work to control the Pi exchange between cytoplasm and mitochondria [55]. These expression changes suggest that there are two prime targets for Pi homeostasis in pineapple D-leaves. Since, the MPTs were only differentially expressed between CN vs. MD-2 as well as CN vs. BL, thus, these three pineapple varieties might be using both types of transporters for Pi homeostasis. In particular to this observation, these three varieties should be explored in future studies. Earlier studies have indicated the involvement of multiple TFs in Pi transport and metabolism in plants. For example, WRKY42, WRKY45, and WRKY6 (along with other unknown TFs) are known for transcriptional regulation of PHTs [56]. Similarly, it is known that MYB TFs regulate the expression of phosphate transport related genes [57, 58]. Our observations that the D-leaves of pineapple varieties differed in the expression of a large number of WRKY, MYB, and bHLH TFs proposes that the pineapple PHTs are being transcriptionally regulated by these TFs [16, 17]. The roles of individual TFs in Pi transport in pineapple e.g., WRKY2, WRKY22, WRKY33, WRKY51-like, and WRKY52 needs specific characterization studies.

The metabolite enrichment in the flavonoid biosynthesis, secondary metabolite biosynthesis, valine and isoleucine biosynthesis is in accordance with the earlier reports that changes in Pi concentrations in leaves can affect the amino acid and flavonoid biosynthesis in tea plants [59]. Earlier studies also revealed that the N, P, and K contents in leaves are associated with the valine contents [60]. This is in line with our observations both in case of variety comparisons based on significantly higher N% (BL) and P% (CN) as discussed above. Taken together, it could be proposed that the studied pineapple varieties differ in their D-leaf P% content due to differential expression and regulation of Pi transport and associated pathways.

### K^+^ transport and metabolism in D-leaves of pineapple

K^+^ is transported in plants with the help of low affinity and high affinity transporters. The KUPs are low affinity K^+^ transporters and uptake K^+^ into the cell when pH is low [61, 62]. The differential regulation of a large number of KUPs in D-leaves of the studied pineapple varieties emphasize that these varieties use KUPs for maintaining the cellular K^+^ levels. Since, the K^+^ influx is always accompanied by decrease in H^+^ efflux [63], thus, the differential regulation of H^+^-ATPases, F-type H^+^-ATPases, and V-type H^+^-ATPases is understandable (Fig. 6). Since K^+^ mainly accumulates in vacuole due to pH gradient [15], and therefore, the expression of genes associated with vacuolar pH gradient development and K^+^ accumulation is an indication of K^+^ levels in leaves. While KUPs allow the transport of K^+^ into the cell, the K^+^ channels (SKOR-like, AKT, KOR, KAT, and TPC) help plants to modulate the K^+^ levels [64] e.g., the expression of SKOR is modulated by the external K^+^ levels. The differential regulation of these K^+^ channels in the D-leaves of the studied pineapple varieties indicated their essential roles in K^+^ homeostasis. Additionally, the CNGC have also the potential to transport K^+^ [65], thus it is obvious from their expression that they are playing similar roles in pineapple D-leaves. The differential expression of *HKT6* in CN, TN4, TN21, and MD4 suggests long-distance K^+^ transport in pineapple leaves. The HKT gene family members have been characterized for their involvement in K^+^/Na^+^ regulation. The HKTs apart from transport, are also involved in mediating salt tolerance in different plants. The presence and the expression of *HKT6* should be specifically studied in pineapple under salt stress conditions [66]. Since, K^+^ transport and accumulation are also associated with Ca^2+^ signaling therefore, the changes in the expression of related genes are important observation [67]. Particularly, it is noteworthy that a large number of Ca^2+^/H^+^ antiporters were differentially expressed in the D-leaves of pineapple varieties, indicating potential roles in K^+^ homeostasis [68]. The large number of Ca^2+^ signaling related proteins were expressed between the pineapple varieties is in accordance with the recent findings. Particularly, it is known that GORK are phosphorylated by CDPK genes [69]. Though we didn’t observe the differential regulation of GORKs, the differential regulation of nine CDPKs implies that in pineapple, they might be interacting with other K^+^ channels or probably GORKs. We say this because we also noted the varied expression of PP2Cs (Fig. 6) and PP2CAs (Supplementary Table 2). PP2CA in Arabidopsis is known to interact physically with GORK and inhibits its current [70]. Thus, the CKPK and PP2CAs together regulate GORK. A further characterization of this type of K^+^ regulation in pineapple would reveal the possible causes of no GORK differential expression. Other than these, studies have reported evidences that low K^+^ signaling is regulated by Ca^2+^-dependent pathway involving integrin-linked kinase (ILK), HAK5, and CMLs. Our findings in transcriptomic analysis showed the differential expression of CMLs (Fig. 6) and ILKs (Supplementary Table 2) suggesting that K^+^ transport in pineapple D-leaves is being Ca^2+^-dependent. From the metabolite perspective, our results that highly accumulated DAMs were enriched in citrate cycle are relevant to earlier reports that changes in K^+^ levels cause the differential accumulation of metabolites e.g., citrate [71]. Additionally, the increased accumulation of lipids supports earlier results that K^+^ content variation in plants affect lipid metabolism [72]. Taken together, it can be stated that the studied pineapple varieties have different K% contents in their D-leaves and its transport and homeostasis are being regulated by known K^+^ transporters and channels under the influence of Ca^2+^ signaling.

## Methods

### Plant Materials and growth conditions

Seven pineapple varieties i.e., Golden pineapple (MD-2), Bali (Comte de Paris, BL), Caine (CN, also known as Smooth Cayene), Tainong 17 (T17), Tainong 21 (T21), Tainong 4 (T4), Tainong 23 (T23) were obtained from Key Laboratory of Tropical Fruit Tree Biology, Ministry of Agriculture, The South Subtropical Crops Research Institute of Chinese Academy of Tropical Agricultural Sciences. No permissions are necessary to use such samples. The formal identification of the samples was conducted by Prof Jing Chen and no voucher specimens have been deposited. The plant materials were planted in Xuwen QuJie Town, Zhanjiang, Guangdong Province in November 2019. The soil was composed of laterite developed from basalt. Three well-grown pineapple plants (11 months old) of each genotype were randomly chosen for the leaf harvesting (October 2020). Three biological replicates were chosen from each variety. 5-10 cm long pineapple D- leaf fragments (fresh weight about 20-30 g) were collected. The fragments were cut from the middle of pineapple D-leaf using scissors. The leaves were further cut into smaller pieces, mixed well, and placed in a 15 ml centrifugation tube. Centrifugal tubes were stored in a plastic foam container having liquid nitrogen. The samples were then taken back to the laboratory and stored in a -80 cryogenic chamber for the determination of N, P, and K contents, detection of metabolites, and transcriptome analysis.

### Determination of N, P, and K contents in D-leaves

The N, P, and K contents of the D-leaves of each variety were measure in triplicate as reported earlier [73]. The samples were digested in concentrated H_2_SO_4_-H_2_O_2_. N, P, and K were measured using azometer, spectrophotometer, and a flame photometer, respectively [74].

Differences among the varieties regarding individual N, P, and K contents were assessed considered significant at p < 0.05. The statistical analyses were done in Microsoft Excel ® 2019.

### Transcriptome analysis

#### RNA extraction, library construction, and sequencing

Total RNA was extracted using Spin Column Plant total RNA Purification kit (Sangon Biotech, Shanghai, China) and the integrity and concentration of RNA was determined using agarose gel electrophoresis and Qubit 2.0 Fluorometer, respectively. Library preparation, quality inspection, and sequencing was done as reported earlier [75].

### Bioinformatic analyses of the sequencing data

High quality reads were filtered to ensure the accuracy of the downstream analyses. To this regard, reads with adapters were removed, removing the paired readers having N content > 10 %, and if the number of low-quality (Q ≤ 20) bases in any reads is >50 % of the bases of the reads. Following this, we determined the sequencing error rate and GC content distribution and the output of sequencing was presented in Microsoft Excel ® 2019.

The clean reads were compared to the reference genome [76] using HISAT2 [77] and the results were presented in Microsoft Excel ® 2019. The gene expression was then quantified as Fragments Per Kilobase of transcript per Million fragments mapped (FPKM) and the distribution of gene expression in each replicate of each variety were presented as expressive box plot. Pearson Correlation Coefficient (PCC) and principal component analysis (PCA) between the expression of varieties and replicates was computed in R using prcomp (www.r-project.org).

The count of the reads was computed using featureCounts followed by Benjamini-Hochberg method to obtain false discovery rate (FDR). Based on the screening criteria i.e., |log2 Fold Change| >= 1, and FDR <0.05, the total number of differentially expressed genes (DEGs), number of up/down-regulated genes in each comparison were counted. The graphs and plots for the expression data were computed in Corset (https://code.google.com/p/corset-project/). The DEGs were then functionally annotated and enrichment analysis was performed according to KEGG (Kyoto Encyclopedia of Genes and Genomes (https://www.genome.jb/kegg) [78].

### Quantitative RT-PCR analysis

The expression of selected DEGs was evaluated using the qRT-PCR analysis. The *Actin* gene was used in this experiment as the internal control to standardize the expression results [74]. The PCR reactions and subsequent analysis of the expression data were carried out as reported earlier [74] on a Rotor-Gene 6000 machine (Qiagen, Shanghai, China) using primers designed in Primer 3 (Table 1).

**Table 1:** List of Primers used in the qRT-PCR analysis

Gene ID	Primer forward sequence (5’-3’)	Primer reverse sequence (5’-3’)
***Actin***	CGAAATACCATTGAGC	TCCACTGTATTTCTCGCC
***gene-LOC109705186***	AGGAGAAAAGGAGTACC	AGCCACTATACTGCAT
***gene-LOC109706543***	ACTACGCCTATTGAGACCG	CGAGAAGGATTTGACCTA
***gene-LOC109706890***	CGAGTGCTAACATTTCTT	TTGCCCCATAAAACCTC
***gene-LOC109713520***	GAAAGGAAGAAACGCTT	AATGAGGCCAAAACACAAT
***gene-LOC109714246***	TTCGTTGAGAAACCTACG	TCAATCACTTCCATCTCA
***gene-LOC109714540***	GATTGAGAGGAGGCAAG	TCTATCACTTCCCTCTTTT
***gene-LOC109721922***	GTGATCTACGTGCGAC	TGGATATACCCCATCTAT
***gene-LOC109706251***	GAACCACTTACGCCTTAC	TTTTCCTGTTTGGTATC
***gene-LOC109722874***	AATTGAGCCGTATGAGGTA	TTACGGTGCCTACCAAT
***gene-LOC109710899***	TGGACAAAGACACAATC	TTGAATTTCGATCTTTCC
***gene-LOC109725533***	CATGCAAAAACTGCCAAA	AGCACTCAAAGGTTCAAT
***gene-LOC109707144***	TGGTTCATACATCCTTGG	TAATTGATGCCTAAATTT
***gene-LOC109714021***	CTGCCCAATATTTGAGTT	ACCTCCTCCCTACACAA
***gene-LOC109722013***	AACCTCTTTCGACGA	GATTTTGACACCCCTGA
***gene-LOC109715538***	TAACTCGTCCCGAAAACACC	TCGATGAATGGCAACGATAA
***gene-LOC109709647***	GATTACCACGTACAATCGGA	TAGAGCCATGGACCGCTAAC
***gene-LOC109717511***	CAAAGCCTTTACGCAAGGAG	TAATTGGCCCCTTCAACAAG
***gene-LOC109712027***	GGCTGAAGGGAGGAAA	ATCGTGGTCGTTGTCG
***gene-LOC109711673***	TGGAACCTGCTACATTGCTG	CATTTTGCCAGGTTCAAGGT
***gene-LOC109722589***	GGTGGACAGCCAGATT	CATGTCTGGCAACAAAATCG
***gene-LOC109715646***	GGATGGTCCTTGTCAAACTCA	TTGGCCATGTTGTCACTTGT
***gene-LOC109704483***	GATAATGGCGAATGGCTGAT	TCATCCCTGGAATGTCGAGT
***gene-LOC109706574***	GTAAGCAAGTGGCGCATACA	AAGACGGGTGGAGAAA
***gene-LOC109727419***	GAAGCTCAGCAGCATCAGAA	TGGGTTCTATGGCAAAGGAT
***gene-LOC109724194***	ACAGCGTTCTGAAAGCAACC	AGCCAGTGGATCCATTTCAC
***gene-LOC109723665***	CACTCACCCAGAATGGCTTT	TCGGTGGACCTTATTG
***gene-LOC109710029***	TCTATGACTTGGAGGAACA	CTGTCGGACGAGGATT
***gene-LOC109722805***	AGTACGGCAAGGGACACATC	ACGTGAGCAAACCCCTTTTT
***gene-LOC109704450***	TGCTGGAACTACTGACG	GCCCAAGCTTTAATGCCATA

### Widely targeted metabolomics

#### Detection of metabolites

Pineapple D-leaf samples were freeze-dried in a vacuum freeze dryer (scientz-100 F) and crushed in a mixer mil (MM400, Retsch). A 100 mg of lyophilized powder was mixed with 1.2 ml of 70 % methanol, vortexed for 30 s after every 30 min for six times and stored at 4 ℃ overnight. The samples were centrifuged at 12,000 rpm for 10 min, the extracts were filtered (BLAA-104, 0.22 μm) and processed for UPLC-MS/MS analysis. The UPLC conditions and ESI-Q TRAP-MS/MS analyses were done according to earlier reports [75].

### Data analysis

The PCA on metabolite detection results was performed in R using prcomp. The hierarchical cluster analysis and PCC were computed in R using pheatmap. For HCA, normalized signal intensities of metabolites (unit variance scaling) are visualized as a color spectrum.

Significantly regulated metabolites between groups were determined by VIP (Variable Importance Projection) >= 1 and absolute Log2FC (fold change) >= 1. VIP values ​​were extracted from OPLS-DA result, which also contain score plots and permutation plots, was generated using R package MetaboAnalystR. The data was log transform (log2) and mean centering before OPLS-DA. In order to avoid overfitting, a permutation test (200 permutations) was performed.

The identified metabolites were annotated in KEGG and the pathways having significantly accumulated metabolites were fed into metabolite sets enrichment analysis and their significance was determined by hypergeometric test’s p-value.

## Supplementary Information

**Additional file 1.**

**Additional file 2.**

## Data Availability

The raw transcriptome data has been submitted to NCBI SRA under the project number: PRJNA739322.

## Abbreviations

DAM: differentially accumulated metabolites
DEG: differentially expressed gene
FPKM: fragments per kilobase of exon model per million reads mapped
FDR: false discovery rate
GO: gene ontology
K: potassium
N: nitrogen
P: phosphorus
KEGG: Kyoto Encyclopedia of Genes and Genomes
PCA: principal component analysis
qRT-PCR: quantitative real-time PCR
TF: transcription factor
