# Supplementary material for: Integrative transcriptomic and metabolomic analysis of D-leaf of seven pineapple varieties differing in N-P-K% contents
Source: BMC Plant Biol. 2021 Nov 22;21:550. doi: 10.1186/s12870-021-03291-0 (PMC8607640; doi:10.1186/s12870-021-03291-0)
Supplement: Supplementary file 1 — Additional file 1. [file 12870_2021_3291_MOESM1_ESM.docx]

**Supplementary figure 1.** Cumulative nutrient content (N+P+K %) in studied pineapple varieties.
